# Food consumption, nutritional intakes and the role of milk formulas in nutrient adequacy among young children from birth to 2 years living in urban Algeria

**DOI:** 10.1017/S1368980022000957

**Published:** 2022-04-22

**Authors:** Alice Busnel, Marine Domet, Khaoula Ramchani Ben Othman, Caroline Desclée De Maredsous, Hanane Ghomari-Boukhatem, Malika Bouchenak, Karim Bouziane-Nedjadi, Peggy Drouillet-Pinard

**Affiliations:** 1 Université Paris-Saclay, AgroParisTech, INRAE, UMR PNCA, Paris, France; 2 Danone Nutricia Research, R.D. 128 - 91767 Palaiseau Cedex, France; 3 Laboratoire de Nutrition Clinique et Métabolique, Faculté des Sciences de la Nature et de la Vie, Université Oran1, Oran, Algeria; 4 Faculty of Medicine, Oran, Algeria

**Keywords:** Algeria, Infants, Breast-feeding, Nutrition, Milk formulas

## Abstract

**Objective:**

Undernutrition, stunted growth and obesity remain a concern in Algeria. Currently, limited data are available on nutrient intakes among children. Our study aimed to describe food and nutrient intakes and the role of milk formulas among Algerian children.

**Design:**

Dietary intakes were collected using a 4-d interview-based survey for children aged 0–24 months, living in urban areas in Algeria in 2019.

**Setting:**

Food consumptions were described. For children aged 6–24 months, nutrient intakes and adequacy were estimated. Modelling was used to estimate the nutritional impact of substituting cow’s milk for age-appropriate infant formulas (IF).

**Participants:**

Totally, 446 children aged 0–24 months.

**Results:**

Before 6 months, 91·6 % of infants were breastfed. Breastmilk was also the main milk consumed between 6 and 12 months, whereas cow’s milk predominated after 12 months. In children aged 6–24 months, nutrient adequacy prevalence was above 75 % for the majority of nutrients. However, less than 30 % of the children had adequate intakes for total fats, Fe and vitamin D. Simulated substitution of cow’s milk for IF led to improved adequacy for proteins, Fe, and vitamins D and E.

**Conclusions:**

Our study showed that breast-feeding rates were high until 6 months, then declined with age. Consumed foods allowed Algerian children aged 6–24 months to meet most of their nutritional needs, but inadequate intakes were reported for some key nutrients. Our modelling suggested that milk formulas may help to improve nutrient adequacy among non-breastfed infants. Other dietary changes could also be further investigated to enable children to meet all nutritional recommendations.

Infancy and early childhood are both periods of rapid growth and development and are therefore associated with specific nutritional needs. Indeed, nutrient requirements/kg of body weight are proportionally higher during these stages than at any other time in the life cycle. For instance, the human brain almost triples in weight from birth to 3 years of age, by which time it has reached 85 % of its adult size^(1)^. Micronutrients, such as Fe and vitamin D, are particularly crucial for brain development and normal growth, with inadequate intakes being associated with cognitive impairment^(2,3)^, skeletal deformities^(4)^ and an increased risk of respiratory tract infections^(5)^.

The WHO recommends exclusive breast-feeding for infants until at least 6 months of age^(6)^. In 2005, the WHO developed a set of nine guiding principles for the feeding of non-breastfed children aged 6 to 23 months^(7)^, addressing the amount of food needed and food consistency, meal frequency and energy density, as well as nutrient requirements. While many of these recommendations remain relevant for the initial aim of preventing undernutrition, these guidelines are now due to be updated, using more recent evidence from worldwide studies, to take into account rising rates of childhood obesity and the prevalence of diet-related non-communicable diseases^(8)^.

Indeed, despite WHO recommendations, anaemia and undernutrition, together with the added burden of obesity, are known to be prevalent in Algeria. According to recent national studies, 10 % of Algerian children aged under 5 years have stunted growth^(9,10)^. Undernutrition is most prevalent in the southern part of Algeria, where poverty rates are higher and where access to health services is more limited. At the same time, the proportion of people who are overweight or obese is increasing among the Algerian population as a whole, reportedly affecting 13 % of children under 5 years old in 2019^(10)^ and 27 % of adults in 2017^(9)^. Micronutrient deficiencies are also common. The prevalence of anaemia is high, with approximately half of cases being due to Fe deficiency among women of childbearing age^(11)^, and rates of 30 % among children under 5 years old, and of 36 % among women of reproductive age (between 15 and 49 years)^(12)^. Finally, vitamin A deficiency is known to be prevalent in the south of the country^(13)^.

Literature about the nutritional status of Algerian children is scarce, and there have been no recent studies describing the dietary habits and nutrient intakes of Algerian infants and children aged between 0 and 2 years. At present, there are also no data about the prevalence of nutrient adequacy in this young Algerian population. The present study aimed to describe the major food groups consumed by children from birth to 24 months living in urban regions of Algeria, and to evaluate nutritional intakes and adequacy prevalence among children aged from 6 to 24 months. We also studied the impact of milk formulas on nutrient adequacy in this population, in particular the roles of a follow-on formula (FOF) developed for infants aged 6 to 12 months and a young child formula (YCF) developed for children aged 1 to 3 years.

## Methods

### Study design and setting

This observational study was conducted using data from an interview-based, subnational, dietary survey carried out in Algeria between January 2019 and May 2019. To ensure data quality and reliability, all interviewers received instructions and training for recruitment and data collection.

### Survey population and sampling strategy

The target survey population was a sample of infants and toddlers (aged 0 to 2 years) living in urban areas of Algeria: Setif (northeast), Oran (west), Algiers (centre) and Djelfa (Saharan Atlas). Children were considered ineligible if they had parents, guardians, family members or close family friends who had participated in any other research study during the previous 6 months to avoid burden related to the questionnaires, or who worked in marketing, market research, advertising, journalism and communication, or in the retail or manufacture of dairy products to avoid bias in the declaration and/or consumption of Danone categories products. The final survey sample was selected using a three-stage sampling strategy (based on sampling area, and household and individual factors), with quotas being defined to ensure that parents or caregivers from different socio-economic classes (SEC), household sizes, age groups and sexes were represented across regions. Potential participants were identified at random through door-to-door contact. In addition, weighting factors were applied to demographic characteristics (age, city and SEC category) for each participant to ensure the representativeness of our sample.

### Evaluation criteria

Evaluation criteria included descriptive analyses of food consumption for the whole study population, and, in children aged 6 to 24 months, assessment of nutrient intakes, the prevalence of nutritional adequacy, and the impact of milk formulas on nutrient intakes and adequacy prevalence. Criteria were assessed separately for infants aged >6–12 months and toddlers aged >12–24 months.

### Data collection

#### Socio-economic class

Participants were classified as belonging to a SEC (ranging from high to low: A, B, C1, to C2) based on the occupation status of the head of the family, type of accommodation and possessions (washing machine, microwave, number of cars, etc.).

#### Weight and height

The height and weight of the participants were reported by caregivers during the first survey interview. To limit bias in the assessment of the weight status based on reported values, data below the 1st or above the 99th percentiles of the WHO child growth standards^(14)^ were excluded. Age-specific and sex-specific BMI *Z*-scores were then calculated, based on the WHO child growth standards for children aged 0 to 2 years^(15)^, using the WHO R package^(14)^.

#### Dietary data

Quantitative dietary data were collected during face-to-face interviews carried out during four home visits. The data were recorded in a 4-d food diary, covering 3 consecutive weekdays and 1 d during the weekend. During the interviews, parents or caregivers were asked, with the help of a paper food diary, to recall all food and drink given to their child in the previous 24 h before the visit. Portion sizes were estimated using the photographic atlas of food portions prepared for the Emirate of Abu Dhabi nutritional survey^(16)^ and using household measures (glasses, cups, etc.) or were provided directly as a weight, volume or standard unit (such as a commercial food portion).

For breastfed children, WHO estimates of the mean quantity of breastmilk produced per d for developing countries^(17,18)^ were attributed to each child according to their age. USDA values^(19)^ were used for breastmilk composition. Information on whether the participants had received micronutrient supplements was also collected. However, as no information regarding the characteristics or quantities of the supplements was recorded, these data were not included in the analysis.

#### Assessments of dietary and nutrient intakes and nutrient adequacy

Dietary intake (in g/d or ml/d) was assessed from the data recorded in the 4-d food diary after the classification of the consumed foods into 28 groups. Milk formulas included infant formula (IF) adapted for children from birth to 6 months, FOF from 6 to 12 months and YCF from 1 to 3 years.

Macronutrient and micronutrient intakes were then evaluated for children above 6 months of age using food composition databases^(20,21)^, Danone data for infant milk formulas and baby cereals, and a nutritional survey tool^(22)^ for traditional recipes. The prevalence of adequate intakes for each nutrient, defined as the percentage of children meeting requirements, was determined according to estimated average requirements (EARs)^(23)^, or according to adequate intake (AI) values in the case of nutrients for which an EAR was not available, using values provided by the European Food Safety Agency (EFSA)^(24)^ and Institute Of Medicine^(25)^ for micronutrients, and the French Agency for Food, Environmental and Occupational Health and Safety (ANSES)^(26)^ for macronutrients, as the European recommendations are those in use in Algeria. Adequacy prevalence was not investigated for the different types of dietary fat as the current EFSA recommendations suggest reducing SFA intakes as much as possible during childhood^(24)^.

For total fats, proteins and carbohydrates, participants were considered as having adequate intakes if their nutritional intakes were within the acceptable macronutrient distribution range (AMDR). For all other nutrients, intakes were considered as adequate if they were above recommended EAR or AI values. When the recommendation for a nutrient was given as a percentage of the energy requirement (E %), intakes were assessed according to the recommended energy requirements for each child depending on their age and sex. For nutrients with a recommended tolerable upper limit (UL), the percentage of individuals with intakes above the UL was also assessed.

#### Assessments of the impact of milk formulas on nutrient intake

The impact of FOF and YCF on nutritional adequacy was first studied by assessing the adequacy prevalence of nutrients between consumers and non-consumers of FOF for infants aged 6–12 months, and between consumers and non-consumers of YCF for toddlers aged 12–24 months. A further analysis was then conducted using simulations involving the quantitative substitution of all milks (cow’s milk and age-inappropriate IF) in non-consumers of FOF or YCF by the same quantity of age-appropriate FOF or YCF. No substitutions for breastmilk were included in any of the simulations.

#### Sample size and bias limitation

Following the 2009 EFSA guidelines^(27)^, a minimum sample population of 130 infants in each age group was needed to assume a significance level of 1 % for the 95^th^ percentile. Thus, 506 children aged from 0 to 24 months were included in the survey population. As the methodology recommended by the EFSA^(28)^ was not appropriate for identifying outliers among children under 1 year of age or among those being breastfed, underreporting and overreporting were assessed using the 5^th^ and 95^th^ percentiles for energy intake in each age group. Participants with implausible intakes or energy intakes outside of this range were excluded (*n* 60).

#### Statistical analyses

All statistical analyses were performed using R software (version 3.6.1), taking into account individual weighting factors. The limit for statistical significance was set at *P* < 0·05. Data on participant characteristics, and food and nutrient intakes, and on the prevalence of nutritional adequacy are expressed as means and standard deviation or percentages. Across age groups, Rao–Scott chi-square tests were used to assess the distribution of the participants for sex, SEC and regions, and Student’s *t* tests were used for BMI *Z*-scores. Student’s *t* tests were also performed to compare nutrient intakes between consumers and non-consumers of the different types of milk.

## Results

### Study population

Data from 446 participants were included in the analysis: 100 aged 0 to 6 months, 94 aged 6 to 12 months and 252 aged 12 to 24 months. The characteristics of the analysed sample population are provided in Table 1. The mean BMI *Z*-score for infants aged from 0 to 12 months was in the recommended range (-2, 1)^(14)^. However, children aged over 12 months had a mean *Z*-score of 1·0, indicating that they were at risk of being overweight. Closer analysis of the data on case-by-case basis revealed that among the participants for whom anthropometric data were reported (*n* 225), 54 % had a normal weight status, 17 % were at risk of being overweight, 14 % were overweight, 5 % were obese, 7 % were stunted and 3 % were severely stunted. The most highly represented SEC among caregivers was the lowest class (C2), and the most highly represented sample region was Algiers, followed by Oran. The percentage of breastfed children decreased with age from 92 % before 6 months to 43 % after 12 months.

**Table 1:** Characteristics of the analysed sample population

	Total sample analysed (*n* 446)		(0–6) months (*n* 100)		(6–12) months (*n* 94)		(12–24) months (*n* 252)		*P*-value*
	*n*	%	*n*	%	*n*	%	*n*	%	
Age (months)									
Mean	12·5		3·0		7·9		17·6		–
sd	7·0		1·5		1·5		4·0		
Sex, girls	201	44·0	43	43·1	45	45·3	113	43·8	0·79
BMI† *Z*-score									
Mean	0·3		−0·9		−0·3		1·0		<0·001
sd	1·8		2·0		1·4		1·5		
Weight (kg)†									
Mean	9·7		5·8		8·1		11·4		–
sd	2·7		1·1		1·0		1·7		
Height (cm)†									
Mean	74·6		60·4		69·4		80·7		–
sd	9·5		4·1		3·4		5·6		
SEC‡									
AB	63	10·8	19	15·1	11	9·3	33	9·8	0·41
C1	135	29·5	32	32·1	31	31·5	72	27·9	
C2	248	59·7	49	52·8	52	59·2	147	62·3	
Region									
Algiers	169	47·3	36	44·8	39	51·6	94	46·6	0·83
Oran	106	24·7	27	28·0	19	20·4	60	25·0	
Setif	87	14·4	22	16·4	18	14·2	47	13·8	
Djelfa	84	13·6	15	10·8	18	13·8	51	14·6	
Breastfed, %		60·0		92·0		74·3		43·2	–

SEC, socio-economic class.

* *P*-values were calculated using the Student’s *t* test for age and BMI *Z*-score and using the Rao–Scott chi-square test for sex, SEC and region.

† *n* 225 for the total population, *n* 44 for the 0- to 6-month group, *n* 48 for the 6- to 12-month group and *n* 133 for the 12- to 24-month group.

‡ AB was the highest class, C1 was the middle class and C2 was the lowest class.

### Food consumption

Food consumption across the three age groups is summarised in Table 2. Milk was a major component of the diet for participants from all age groups, accounting 82·7 % of the total quantity of food consumed before 6 months of age and 44·1 % of the food consumed in children aged 12–24 months. Before 6 months of age, 83·0 % of all the milk consumed was breastmilk, with the remaining 17·0 % of milk consumed being mostly IF. Before 6 months, 23·0 % of infants were exclusively breastfed (no other food or liquid intakes were reported), 68·6 % received mixed feeding (breastmilk, milk formula and complementary foods) and 8·0 % had never received breastmilk.

**Table 2:** Average daily food intakes by infants and young children

Food category	(0–6) months (*n* 100)		(6–12) months (*n* 94)		(12–24) months (*n* 252)	
	g or ml/d	% of the daily portion	g or ml /d	% of the daily portion	g or ml/d	% of the daily portion
Appetisers, nuts and seeds	0·0	0·0	0·0	0·0	0·6	0·0
Baby cereals	21·4	2·6	53·4	4·0	19·5	1·2
Baby fruit compote	2·2	0·3	7·7	0·6	3·8	0·2
Baby infusions	17·1	2·1	2·8	0·2	1·8	0·0
Baby savoury dish	0·1	0·0	1·5	0·1	11·7	0·7
Bread and bakery	0·7	0·1	5·1	0·4	17·0	1·1
Breastmilk	572·1	68·8	418·2	31·6	168·7	11·4
Cereals and breakfast cereals	0·1	0·0	0·2	0·0	0·8	0·0
Cheese	0·3	0·0	4·4	0·3	5·7	0·4
Chocolate and confectionery	0·1	0·0	0·1	0·0	3·5	0·1
Cold drinks	30·0	3·6	146·6	11·1	248·9	16·8
Cow’s milks	11·9	1·4	87·8	6·6	349·5	23·6
Dairy desserts and other desserts	0·0	0·0	3·2	0·2	6·5	0·4
Egg and egg dishes	0·2	0·0	3·3	0·3	10·3	0·6
Fish and fish dishes	0·1	0·0	2·6	0·2	5·2	0·3
Fruits	3·3	0·4	24·2	1·8	49·6	3·3
Hot drinks	5·6	0·7	1·3	0·1	4·4	0·3
Meal substitute	0·0	0·0	1·8	0·1	1·2	0·0
Meat and meat dishes	0·0	0·0	3·6	0·3	21·4	1·3
Milk formulas (IF, FOF and YCF)	117·2	14·1	246·6	18·6	134·4	9·1
Pastries, cakes and biscuits	1·1	0·1	7·1	0·5	23·2	1·4
Prepared meals	0·0	0·0	8·3	0·6	42·7	2·9
Sauces, fats and condiments	1·1	0·1	8·4	0·6	16·5	0·8
Soups	21·3	2·5	134·5	10·2	79·9	7·6
Starches	12·8	1·5	51·4	3·9	107·5	7·3
Sugar and sweeteners	6·7	0·8	2·3	0·2	8·9	0·4
Vegetables	0·8	0·1	14·6	1·1	32·2	1·6
Yoghurt and cottage cheese	8·0	1·0	81·1	6·1	105·3	7·1
Total	834·3	100·0	1322·2	100·0	1481·1	100·0

IF, infant formula for infants aged under 6 months; FOF, follow-on formula for infants aged between 6 and 12 months; YCF, young child formula for children aged between 1 and 3 years.

Between 6 and 12 months, breastmilk represented 55·5 % of all milk consumed, whereas after 12 months, cow’s milk was the main type of milk consumed. None of the participants were exclusively breastfed after 6 months. About 25 % of the infants aged 6 to 12 months and 57 % of the toddlers aged 12 to 24 months did not consume any breastmilk.

Milk formulas represented the second major food type consumed before 12 months of age, mainly IF from birth to 6 months and FOF between 6 and 12 months. The intake of cow’s milk increased with age, from about 1·0 % of the total food quantity consumed in infants aged 0–6 months to 24·3 % in toddlers aged 12–24 months. Cold drink consumption, which included mainly water and fruit juice, also increased with age. Soups also formed a considerable part of the diet after 6 months of age.

Solid foods – such as baby cereals, mashed potatoes, cream cheese, fruits, plain biscuits and white sugar – began to be introduced in small quantities (under 4 % of the total food consumed) after the age of 1 month for 4 % of the infants, and half of the infants had been introduced to solid foods at 4 months. After 6 months, vegetables (excluding soups), bread, prepared meals, meat and meat dishes, eggs, fish and pastries were introduced in small quantities. Solid food consumption then increased with age, representing about one-third of the diet for children aged between 12 and 24 months. Complementary foods involved similar foods in both non-breastfed and breastfed children; however, the proportions of solid foods and cow’s milk in the diet were higher for non-breastfed children than for breastfed children.

Feeding patterns were similar across all age groups. Food consumed upon waking and during the night exclusively involved the consumption of milk (breastmilk, milk formulas or cow’s milk). Breakfast time, mid-morning, mid-afternoon, before-dinner and after-dinner food intakes involved the consumption of small quantities of solid foods or beverages.

No considerable differences in food consumption were observed between males and females in any of the age groups. Small differences in food consumption were observed between SEC: compared to those in the lower SEC, infants aged 0–6 months in the highest SEC (AB) did not consume any foods from the pastries-cakes-biscuits group, but they consumed more sugars-sweeteners. In addition, infants aged 6–12 months in the highest SEC consumed more milk formulas and less breastmilk than those in the lower SEC.

### Nutritional intakes

The intake and adequacy prevalence of the thirty nutrients analysed for the children aged 6 to 24 months are shown in Table 3. Nutrient intakes were not studied for infants aged 0–6 months due to the high rate of breast-feeding in this age group and the inherent difficulties of accurately assessing breastmilk intakes.

**Table 3:** Nutritional intakes and adequacy prevalence for infants and young children according to age group

Nutrients	>6–12 months ; *n* 94				>12–24 months ; *n* 252			
	Intakes		Recommendations (AMDR range) AI, EAR (source)	Adequacy prevalence* (%)	Intakes		Recommendations (AMDR range) AI, EAR (source)	Adequacy prevalence*(%)
	Mean	sd			Mean	sd		
Energy (kcal)	939·6	236·0	593·0–708·0‡ (EFSA 2013)	95·7	1122·8	287·2	765·0–987·0‡ (EFSA 2013)	79·4
Fat (g/E %†)	44·4	13·4	45–50 (EFSA 2019)	27·7	42·7	13·4	35–40 (EFSA 2019)	24·2
MUFA (g/E %†)	18·3	8·1	–	–	16·1	7·0	–	–
PUFA (g/E %†)	5·1	1·5	–	–	5·3	2·4	–	–
SFA (g/E %†)	19·1	4·8	–	–	18·5	5·8	–	–
Protein (g/E %†)	27·5	12·3	7–15 (ANSES 2016)	79·8	43·0	15·1	6–15 (ANSES 2016)	54·0
Carbohydrates (g/E %†)	106·0	28·9	40–50 (EFSA 2019)	31·9	138·0	43·4	45–60 (EFSA 2019)	63·5
Fibre (g)	6·0	3·0	6·0 (EFSA 2010)	41·5	8·7	3·1	10·0 (EFSA 2019)	25·8
Ca (mg)	749·8	316·2	280·0 (EFSA 2019)	100·0	882·1	345·1	450·0‡ (EFSA 2019)	90·9
Cu (mg)	0·7	0·5	0·4 (EFSA 2019)	93·6	0·8	0·5	0·7 (EFSA 2019)	55·6
Iodine (µg)	172·8	111·5	70·0 (EFSA 2019)	86·2	217·5	151·3	90·0 (EFSA 2019)	90·1
Fe (mg)	5·2	3·5	8‡ (EFSA 2019)	19·1	5·8	3·3	7·0‡ (EFSA 2019)	25·4
Mg (mg)	109·6	45·1	80 (EFSA 2019)	71·3	178·4	69·0	170·0 (EFSA 2019)	46·0
Mn (mg)	0·9	2·2	0·5 (EFSA 2019)	42·6	1·3	1·2	0·5 (EFSA 2019)	89·7
Phosphorus (mg)	557·6	273·9	160 (EFSA 2019)	98·9	875·3	343·5	250·0 (EFSA 2019)	98·8
Potassium (mg)	1273·5	487·1	750 (EFSA 2019)	81·9	1878·5	641·3	800·0 (EFSA 2019)	98·4
Se (µg)	36·4	15·0	15 (EFSA 2019)	98·9	55·6	23·4	15·0 (EFSA 2019)	100·0
Na (mg)	1574·6	2267·7	200 (EFSA 2019)	94·7	2421·0	3119·3	1100·0 (EFSA 2019)	49·2
Zn (mg)	4·7	2·0	2·4‡ (EFSA 2019)	89·4	5·8	1·9	4·3‡ (EFSA 2019)	75·0
Retinol (µg)	618·3	415·8	190·0‡ (EFSA 2019)	100·0	469·4	401·6	205·0‡ (EFSA 2019)	88·5
Vitamin C (mg)	74·4	29·2	50 (IOM 2000)	76·6	66·7	38·5	15·0‡ (EFSA 2019)	96·4
Vitamin D (µg)	6·3	4·7	10·0 (EFSA 2019)	21·3	4·9	3·8	15·0 (EFSA 2019)	1·6
Vitamin E (mg)	6·4	4·0	5·0 (EFSA 2019)	58·5	6·4	3·7	6·0 (EFSA 2019)	42·5
Thiamine (mg)	0·6	0·3	0·2‡ (EFSA 2019)	94·7	0·8	0·3	0·3‡ (EFSA 2019)	96·8
Riboflavin (mg)	1·1	0·5	0·4 (EFSA 2019)	95·7	1·5	0·6	0·5‡ (EFSA 2019)	97·2
Niacin (mg)	5·1	2·8	3·5‡ (EFSA 2019)	63·8	7·1	3·4	4·5‡ (EFSA 2019)	72·6
Pantothenic acid (mg)	2·8	1·6	3·0 (EFSA 2019)	41·5	4·2	1·7	4·0 (EFSA 2019)	51·6
Pyridoxine (mg)	0·7	0·3	0·3 (EFSA 2019)	87·2	1·0	0·3	0·5‡ (EFSA 2019)	92·1
Folate (µg)	145·1	66·6	80·0 (EFSA 2019)	81·9	188·6	63·3	90·0‡ (EFSA 2019)	95·6
Cobalamin (µg)	2·0	1·9	1·5 (EFSA 2019)	54·3	3·1	2·1	1·5 (EFSA 2019)	83·3

AMDR, acceptable macronutrient distribution range; AI, adequate intake, that is, covers the needs of all healthy individuals in the considered groups (a specific age, sex and life stage) but lack of data or uncertainty in the data prevent specifying with confidence the percentage of individuals covered by this intake; EAR, estimated average requirement, that is,. amount of a nutrient that is estimated to meet the requirement for a specific criterion of adequacy for half of the healthy individuals of a specific age, sex and life stage; RDA, Recommended Dietary Allowance, that is, average daily dietary intake level (sufficient to meet the nutrient requirements of nearly all (97–98 %)) healthy individuals in a group.

* Measure of the percentage of individuals in the sample who meet recommendations for the given nutrient.

† Intakes are in grams and recommendations in energy percentage.

‡ EAR used as recommendation ((AMDR) or AI otherwise).

#### Nutrient intakes in infants aged 6–12 months and toddlers aged 12–24 months

Mean intakes were aligned with the recommended intakes for more than half of the thirty nutrients investigated, with an adequate intake for more than 75 % of the children.

For infants, mean total fats and carbohydrate intakes were, respectively, below and above the AMDR, resulting in adequacy prevalence values of only 27·7 % for total fats and 31·9 % for carbohydrates. For micronutrients, mean Fe, fibre, Mn, vitamin D and pantothenic acid intakes were under recommended AI levels, with adequacy prevalence values of 19·1 %, 41·5 %, 42·6 %, 21·3 % and 41·5 %, respectively.

For toddlers, total fats were outside the AMDR, with an adequacy prevalence of only 24·2 %. For micronutrients, fibre, Fe and vitamin D mean intakes were under AI levels, with respective adequacy prevalence values of 25·8 %, 25·4 % and 1·6 %. About half of the toddlers had adequate intakes for Mg, Na and vitamin E, with adequacy prevalence values of 46·0 %, 49·1 % and 42·5 %, respectively.

#### Nutritional adequacy in consumers and non-consumers of milk formulas

Milk formulas, mainly FOF for infants aged 6–12 months and YCF for toddlers aged 12–24 months, were often the main sources of nutrients. For example, milk formulas contributed to about 40 % of the Fe intake, 40 % of the vitamin D intake and 35 % of the vitamin E intake for children aged 6–24 months. Further analyses showed that milk formula consumers had intakes that were closer to recommended values for several key nutrients compared to non-consumers.

In FOF non-consumers, intakes of fibre, Fe, vitamins C, D, and E, niacin, pantothenic acid and cobalamin were lower than in FOF consumers with on average two times less than children having adequate intakes (Table 4). The main differences between YCF non-consumers and consumers were observed for Fe, vitamins D and E, with YCF non-consumers having notably lower intakes and adequacy prevalence values for these nutrients than YCF consumers (Table 5).

**Table 4:** Observed and simulated intakes and adequacy prevalences for FOF consumers and non-consumers aged 6–12 months

Nutrient	FOF consumers (observed; *n* 45)			FOF non-consumers (observed; *n* 49)			Simulation 1*			
	Intake/d		Adequacy prevalence (%)	Intake/d		Adequacy prevalence (%)	Intake/d		*P*-value†	Adequacy prevalence (%)
	Mean	sd		Mean	sd		Mean	sd		
Energy (kcal)	969·9	231·8	97·9	916·2	242·4	91·1	935·1	256·3	< 0·001	91·4
Fat (g)	44·0	13·1	27·0	45·0	12·9	33·7	46·7	13·7	< 0·001	35·2
MUFA (g)	18·1	7·1		18·5	8·0		19·4	8·3	< 0·001	
PUFA (g)	5·6	1·6		4·7	1·3		5·2	1·4	< 0·001	
SFA (g)	18·6	4·8		19·6	4·9		20·0	5·1	< 0·001	
Protein (g)	30·0	11·0	80·7	25·5	13·6	76·0	24·5	12·3	< 0·001	84·3
Carbohydrates (g)	111·7	26·5	39·0	101·4	30·5	23·5	103·5	31·8	< 0·001	23·7
Fibre (g)	7·1	3·3	56·3	5·0	2·2	28·1	5·7	2·7	< 0·001	40·1
Ca (mg)	901·8	290·5	100·0	616·2	290·9	100·0	618·3	286·8	< 0·001	100·0
Cu (mg)	0·6	0·1	95·2	0·7	0·6	95·3	0·87	0·6	< 0·001	98·5
Iodine (µg)	190·0	99·3	100·0	157·9	122·8	77·4	159·4	121·7	< 0·001	77·4
Fe (mg)	7·6	3·5	36·8	3·1	1·7	1·5	4·3	2·9	< 0·001	13·6
Mg (mg)	115·7	38·7	91·6	105·0	50·8	60·0	99·2	44·9	< 0·001	61·2
Mn (mg)	1·4	3·2	51·4	0·6	0·4	40·7	0·6	0·4	0·99	39·1
Phosphorus (mg)	638·3	250·1	100·0	489·3	281·2	97·5	461·6	235·6	< 0·001	97·5
Potassium (mg)	1414·5	441·4	97·3	1152·5	501·0	71·9	1112·7	433·0	< 0·001	71·9
Se (µg)	37·0	13·6	100·0	36·1	16·7	98·5	33·6	14·6	< 0·001	100·0
Na (mg)	1360·0	1950·1	98·3	1771·0	2623·1	92·3	1757·0	2622·8	0·22	93·8
Zn (mg)	5·8	2·0	100·0	3·7	1·5	78·7	4·0	1·9	< 0·001	78·7
Retinol (µg)	609·4	164·5	100·0	623·6·1	549·5	100·0	683·8	566·9	< 0·001	100·0
Vitamin C (mg)	88·2	28·3	94·6	61·8	24·0	59·4	71·9	27·5	< 0·001	68·6
Vitamin D (µg)	9·7	4·6	39·1	3·3	2·2	2·7	5·0	4·0	< 0·001	10·8
Vitamin E (mg)	9·0	3·7	90·1	4·1	2·7	28·3	5·5	3·9	< 0·001	45·1
Thiamine (mg)	0·7	0·2	100·0	0·5	0·2	89·9	0·5	0·3	< 0·001	89·9
Riboflavin (mg)	1·3	0·4	100·0	0·9	0·6	91·3	0·9	0·5	< 0·001	91·3
Niacin (mg)	6·3	2·6	90·3	4·2	2·7	42·0	4·6	2·9	< 0·001	55·6
Pantothenic acid (mg)	3·7	1·6	65·3	2·1	1·6	18·2	2·2	1·6	0·02	24·8
Pyridoxine (mg)	0·8	0·3	94·6	0·5	0·3	80·0	0·6	0·3	< 0·001	82·6
Folate (µg)	158·4	53·2	94·5	133·7	77·6	73·4	139·3	81·2	< 0·001	73·4
Cobalamin (µg)	2·1	0·9	72·9	2·0	2·6	38·3	1·8	2·5	< 0·001	30·5

FOF, follow-on formula; IF, infant formula; YCF, young child formula.

* Simulation 1: In non-consumers of FOF aged from 6 to 12 months, all cow’s milk, IF and YCF were replaced by FOF in equal quantities.

† *P*-values for the differences between observed intakes and predicted intakes were obtained using the Wilcoxon test.

However, because of the high number of bootstraps which may lead to even small differences between observed and predicted intakes reaching significance, the results of these tests should be interpreted with caution, taking into account the observed differences.

**Table 5:** Observed and simulated intakes and adequacy prevalences for YCF consumers and non-consumers aged 12–24 months

Nutrient	YCF consumers (observed; *n* 61)			YCF non-consumers (observed; *n* 191)			Simulation 2*			
	Intake/d		Adequacy prevalence (%)	Intake/d		Adequacy prevalence (%)	Intake/d		*P*-value†	Adequacy prevalence (%)
	Mean	sd		Mean	sd		Mean	sd		
Energy (kcal)	1005·1	291·2	60·4	1162·4	288·0	83·0	1230·5	319·7	< 0·001	85·4
Fat (g)	40·3	15·2	41·0	43·5	13·3	18·8	50·9	14·7	< 0·001	31·8
MUFA (g)	15·4	7·3		16·4	6·8		20·2	7·5	< 0·001	
PUFA (g)	5·9	2·3		5·1	2·6		7·1	3·0	< 0·001	
SFA (g)	17·1	6·1		19·0	6·1		20·6	6·1	< 0·001	
Protein (g)	36·8	13·9	64·4	45·0	15·8	47·8	40·0	13·4	< 0·001	75·7
Carbohydrates (g)	121·1	33·4	63·1	143·7	45·1	61·7	150·3	45·9	< 0·001	68·1
Fibre (g)	8·2	2·4	19·1	8·9	3·3	28·4	8·7	3·3	< 0·001	27·1
Ca (mg)	757·2	267·4	85·9	923·3	378·3	93·2	840·9	321·2	< 0·001	90·9
Cu (mg)	0·7	0·2	51·0	0·9	0·6	55·1	1·0	0·6	< 0·001	75·9
Iodine (µg)	192·7	154·2	88·9	225·6	151·5	91·0	232·7	150·7	< 0·001	94·1
Fe (mg)	9·1	4·0	64·8	4·6	2·1	12·0	10·4	4·8	< 0·001	75·6
Mg (mg)	146·5	48·2	24·0	189·1	71·1	54·2	164·4	56·8	< 0·001	40·8
Mn (mg)	1·1	1·4	86·9	1·3	1·0	91·6	1·3	1·0	0·99	91·0
Phosphorus (mg)	693·1	224·4	100·0	936·2	359·1	98·5	764·6	247·5	< 0·001	98·5
Potassium (mg)	1611·3	450·0	97·4	1967·9	661·0	98·7	1661·2	471·3	< 0·001	98·1
Se (µg)	43·9	14·4	100·0	59·4	25·0	100·0	52·5	17·6	< 0·001	100·0
Na (mg)	1863·8	3019·9	34·1	2605·0	3158·7	57·7	2555·6	3162·9	< 0·001	53·9
Zn (mg)	6·4	2·5	74·3	5·6	1·8	74·8	7·1	2·6	< 0·001	84·8
Retinol (µg)	492·9	205·9	97·8	461·9	485·2	84·1	654·8	488·5	< 0·001	98·9
Vitamin C (mg)	100·7	42·0	100·0	55·4	31·2	95·5	105·2	45·7	< 0·001	100·0
Vitamin D (µg)	8·4	4·2	6·7	3·7	2·8	0·0	9·2	5·0	< 0·001	13·5
Vitamin E (mg)	9·8	4·1	74·0	5·3	2·9	31·2	11·4	5·9	< 0·001	83·0
Thiamine (mg)	0·8	0·3	96·5	0·8	0·3	96·6	0·9	0·3	< 0·001	97·6
Riboflavin (mg)	1·3	0·5	98·7	1·6	0·7	96·8	1·5	0·6	< 0·001	96·8
Niacin (mg)	8·2	4·1	77·5	6·7	3·3	71·9	8·9	3·7	< 0·001	89·3
Pantothenic acid (mg)	4·4	1·7	55·1	4·2	1·7	50·3	4·8	2·1	< 0·001	64·4
Pyridoxine (mg)	1·0	0·4	94·9	0·9	0·3	91·8	1·0	0·3	< 0·001	93·4
Folate (µg)	182·4	59·8	96·5	187·9	66·8	96·1	213·2	69·8	< 0·001	98·4
Cobalamin (µg)	2·6	1·6	80·3	3·3	2·4	83·5	2·9	2·2	< 0·001	83·4

FOF, follow-on formula; IF, infant formula; YCF, young child formula.

* Simulation 2: In non-consumers of YCF aged from 6 to 12 months, all cow’s milk, IF and FOF were replaced by equal quantities of YCF.

† *P*-values for the differences between observed intakes and predicted intakes were obtained using the Wilcoxon test.

However, because of the high number of bootstraps which may lead to even small differences between observed and predicted intakes reaching significance, the results of these tests should be interpreted with caution, taking into account the observed differences.

Overall, milk formula consumers had higher Fe, vitamin D and vitamin E intakes than consumers of unfortified cow’s milk.

#### Impact of simulated milk substitutions on nutrient intake

In the first simulation, which involved non-consumers of FOF aged 6 to 12 months, all milk (except breastmilk) was replaced by FOF (Table 4). This resulted in an improvement in mean Fe, and vitamin D and E intakes, and to a lesser extent in protein, fibre and niacin intakes. Indeed, higher adequacy prevalence values were observed for these nutrients: vitamin E (+Δ 16·8 %), niacin (+Δ 13·5 %), Fe (+Δ 12·2 %), fibre (+Δ 12·0 %) protein (+Δ 11·3 %) and vitamin D (+Δ 8·2 %).

In the second simulation, which involved non-consumers of YCF aged 12 to 24 months, the consumption of all other types of milk (except breastmilk) was replaced by YCF (Table 5). This substitution led to improvements in the intake of protein, total fats, Fe, and vitamin D and E. Adequacy prevalence values improved by 28·0 % for protein, 13·0 % for total fats, 63·7 % for Fe, 13·5 % for vitamin D and 51·8 % for vitamin E. Improvements in adequacy prevalence were also observed for Cu, Zn, retinol, niacin and pantothenic acid (+Δ 20·8 %, +Δ 10·0 %, +Δ 14·8 %, +Δ 17·6 % and +Δ 14·1 %, respectively). Mg was the only nutrient analysed for which the substitution led to a fall in adequacy prevalence (-Δ 12·5 %).

Overall, the simulations indicated that the consumption of age-appropriate milk formulas by infants led to improved mean intakes and adequacy prevalence values for proteins, vitamins D and E, and Fe, compared to the consumption of unfortified cow’s milk or age-inappropriate milk formulas.

To identify any potential safety risks, modelled intakes were also compared to recommended UL values (see online Supplemental Tables 1 and 2). This analysis showed that Zn, Cu, retinol and niacin intakes could further exceed UL values after switching to the tested milk formulas.

## Discussion

The present study is the first dietary survey to evaluate the prevalence of nutrient adequacy in a representative sample of children (aged 6 months to 24 months) living in urban areas of Algeria. Our analysis suggests that young children in urban Algeria have an unbalanced diet with ‘empty’ calories: despite the fact that energy intakes were often above EAR values, inadequate intakes of vitamins and minerals were identified, most notably insufficient intakes of vitamin D and Fe. Moreover, 10 % of children in our study were stunted and 19 % were overweight, compared to the rates of 10 % and 13 % observed nationally^(10)^. Our modelling analysis indicated that consuming FOF or YCF could improve protein, Fe, vitamin D and E intakes, suggesting that milk formulas could play a role as part of the strategy to improve nutrient adequacy in children living in urban Algeria. However, the fortification level of Zn, Cu, retinol and niacin in these milk formulas may need to be reduced to take into account actual overall dietary intakes and avoid the risk of exceeding UL recommendations. Our study also highlighted the value of using a theoretical approach to study the effect of diet substitutions, particularly in cases when it may be unacceptable or unfeasible to assess such dietary changes in a real-life setting.

Infant milk feeding practices have been shown to strongly depend on the social, health, economic and educational background of the parents^(29)^. Overall, in our study population, more than 90 % of the infants were breastfed, either exclusively or non-exclusively before 6 months. This compares to rates of 77 % for non-exclusive breast-feeding in infants aged under 5 months and of 87 % for infants breastfed at least once reported previously in a national study^(10)^. In our study, only 23 % of the infants were fed according to the WHO recommendation, the followed guidelines in Algeria, for exclusive breast-feeding during the first 6 months of life^(7)^. Breast-feeding rates in our population declined with age, falling to 71 % between 6 and 12 months, and to about 40 % after 12 months. After 6 months, infants from highest SEC consumed more milk formulas and less breastmilk in our study. Even if the sample size in each SEC is reduced, this observation may highlight working mothers’ difficulties to pursue breast-feeding after her maternity leave (lack of time or lack of workplaces facilities) as described in a neighbouring country^(30)^.

In addition to the WHO recommendation that infants are exclusively breastfed until at least the age of 6 months, additional guidelines recommend that cow’s milk should not be used as the main drink for infants aged under 12 months^(31)^, and that solid foods should be initiated no earlier than the beginning of the fifth month after birth^(32,33)^. In our study, the introduction of complementary foods was reported to have occurred earlier than recommended in 5 % of infants, including the introduction of cow’s milk, soups, infusions and baby cereals, mashed potatoes, yogurt, and biscuits as early as from 1 month of age. A previous study of Algerian infants found that the introduction of complementary foods before 4 months occurred in 14 % of the studied population, with a further 67 % and 19 % of the population being introduced to complementary foods between 4 and 6 months and after 6 months, respectively^(34)^.

For our participants aged between 6 and 24 months, the adequacy prevalence values for Fe and vitamin D were both under 30 %, which is lower than values observed for Fe deficiency in other studies^(13,35)^. However, it should be noted that neither the contribution of sun exposure to vitamin D intake nor the impact of nutrient supplementation were taken into account in our analyses, although only 28 of the 446 infants included in our study were reported to have received supplements. The national policy in Algeria is to provide infants aged 1, 6, 12 and 18 months with two 5-mg doses of oral vitamin D and to provide Fe deficiency testing to allow Fe supplementation to be recommended when needed^(36)^.

Our analyses revealed that infants aged 6–12 months who consumed FOF and children aged 12–24 months who consumed YCF had intakes more in line with recommendations for several nutrients, such as Fe, and vitamins D and E. These results suggest that milk formulas may play a role in helping young children meet the recommended intakes for these key nutrients, especially for infants whose complementary foods do not cover their nutritional needs. This appeared to be confirmed by the improvements in Fe, vitamin D and E intakes observed in our modelling analysis. Similar findings on the impact of YCF on nutritional intakes have been obtained in previous studies carried out on young children living in Western Europe^(37)^, Australia^(38)^, the UK^(39)^ and China^(40,41)^.

Other previous studies have also highlighted that FOF could be used as part of the strategy for combating micronutrient deficiencies^(42,43)^. In contrast, another study found no evidence that formula milk was an important source of dietary Fe in a population of infants aged 6 to 18 months^(44)^.

Results from our study, together with those described in previous reports, have shown that many infants and young children do not have an appropriate level of complementary food in their diet, which can lead to inadequate intakes of several of the key nutrients that can have an impact on growth and development, most notably Fe^(1,3)^ and vitamin D^(4,5)^. The introduction of complementary foods at 6 months of age is often a crucial period for nutrient adequacy. For non-breastfed children, milk formulas could help to improve adequacy in cases where complementary foods are not supplying enough nutrients. Current recommendations and guidelines for health care providers advocate the introduction of Fe-fortified FOF and Fe-rich complementary foods – including meat products and other Fe-fortified foods such as cereals – from the age of 6 months onwards^(31)^. The results of our study, together with those from a previous report demonstrating that the use of fortified milks (FOF and YCF) was effective for supporting the needs of infants who did not transition well onto an appropriate weaning diet^(36)^, highlight how milk formulas can be used as part of a successful strategy to improve the prevalence of nutrient adequacy among infants and young children. Further investigations focusing on increasing intakes of meat or other fortified foods would be necessary to check the acceptability of such changes.

Our study provided a detailed analysis of the dietary and nutrient intakes of a representative sample of infants and young children living in urban Algeria. However, our survey sample did not include infants and young children living in rural areas, and thus, in general, our findings cannot be considered as representative of the whole Algerian population. In addition, although our sample size was sufficiently large for meaningful analysis of the whole population, it may have been too small for analyses of specific subgroups, perhaps explaining why no difference between SEC groups was observed.

Potential bias in the reporting of food intake is a weakness inherent to all methods used to assess food intake. The dedicated training on dietary data collection provided to all interviewers as well as the age of the target population are likely to have limited this bias in our study. However, although children with implausible intakes were excluded from our analyses, the difficulty of quantifying breastmilk consumption and composition may have led to the under- or overestimation of adequacy prevalence values. In addition, the nutrient composition of the milk formulas consumed by the participants in our study was estimated from Danone data rather than from mean compositions of milk formulas from other manufacturers to avoid missing values; however, as the nutrient content of milk formulas is tightly regulated, it is unlikely that any small differences in composition between formula milks made by other manufacturers would have had an impact on the results obtained.

Although our study demonstrated the value of modelling to provide insights into the nutritional impact of dietary changes, more extensive analyses are needed to help improve dietary recommendations. For example, our simulation method was based on the substitution of equal measures (ml for ml) of the different types of milk rather than on isoenergetic substitutions, which would have allowed for the compensation of changes in energy intake^(39)^. Our study also focused only on the roles of FOF and YCF in nutrient adequacy, but other dietary changes could also be considered for improving nutritional intakes in the child population, including evaluations of the effect of introducing fortified foods or completely reorganising diets to optimise intakes. Finally, a more global overview of local food practices and the environment, in particular the costs and physical accessibility of foods, needs to be considered and addressed in future studies. Applying advanced diet modelling, such as the optimisation technique, would allow additional parameters (cost, acceptability, environmental footprint, etc.) to be evaluated for the design of nutritionally adequate and socially acceptable dietary recommendations.

In conclusion, our study highlights the need for additional measures to improve nutritional adequacy among Algerian infants and children and suggests that milk formulas could play a role in improving some key nutrient intakes in this population.

## Supplementary material

For supplementary material accompanying this paper visit https://doi.org/10.1017/S1368980022000957. click here to view supplementary material
