# Supplementary material for: Food consumption, nutritional intakes and the role of milk formulas in nutrient adequacy among young children from birth to 2 years living in urban Algeria
Source: Public Health Nutr. 2022 Apr 22;26(1):171–81. doi: 10.1017/S1368980022000957 (PMC11077457; doi:10.1017/S1368980022000957)
Supplement: Supplementary file 1 [file S1368980022000957sup.zip › S1368980022000957sup001.docx]

### Supplemental Table 1. Observed and simulated risks of excessive intakes for infants aged 6-12 months

| Nutrient | UL | Total infants 6-12 months  % above UL | FOF consumers  % above UL | FOF non-consumers  % above UL | Simulation 1  % above UL |
| --- | --- | --- | --- | --- | --- |
| Calcium (mg) | 1000.0 | 4.3 | 7.7 | 2.7 | 0.0 |
| Iron (mg) | 40.0 | 0.0 | 0.0 | 0.0 | 0.0 |
| Selenium (µg) | 60.0 | 7.4 | 7.1 | 9.2 | 4.5 |
| Sodium (mg) | 1500.0 | 17.0 | 19.9 | 20.1 | 20.1 |
| Zinc (mg) | 5.0 | 38.3 | 63.8 | 15.9 | 27.3 |
| Retinol (µg) | 600.0 | 36.2 | 51.2 | 24.5 | 34.1 |
| Vitamin D (µg) | 38.0 | 0.0 | 0.0 | 0.0 | 0.0 |

**Abbreviations**: FOF, follow-on formula; UL, upper limit defined according to European Food Safety Authority values.

### **Supplemental Table 2. Observed and simulated risks of excessive intakes for toddlers aged 12-24 months**

| Nutrient | UL | Total toddlers 12-24 months  % above UL | YCF consumers  % above UL | YCF non-consumers  % above UL | Simulation 2  % above UL |
| --- | --- | --- | --- | --- | --- |
| Calcium (mg) | 2500.0 | 0.0 | 0.0 | 0.0 | 0.0 |
| Copper (mg) | 1.0 | 22.6 | 16.0 | 21.7 | 29.0 |
| Iodine (µg) | 200.0 | 37.3 | 24.7 | 42.2 | 45.2 |
| Iron (mg) | 40.0 | 0.0 | 0.0 | 0.0 | 0.0 |
| Magnesium (mg) | 65.0 | 99.6 | 100.0 | 99.7 | 99.4 |
| Manganese (mg) | 2.0 | 12.3 | 6.8 | 13.8 | 12.7 |
| Phosphorus (mg) | 3000.0 | 0.0 | 0.0 | 0.0 | 0.0 |
| Selenium (µg) | 90.0 | 9.5 | 1.6 | 13.9 | 1.7 |
| Sodium (mg) | 1500.0 | 30.6 | 24.0 | 37.7 | 36.0 |
| Zinc (mg) | 7.0 | 25.0 | 39.9 | 20.8 | 51.3 |
| Retinol (µg) | 600.0 | 15.1 | 31.0 | 11.9 | 42.9 |
| Vitamin C (mg) | 400.0 | 0.0 | 0.0 | 0.0 | 0.0 |
| Vitamin D (µg) | 63.0 | 0.0 | 0.0 | 0.0 | 0.0 |
| Vitamin E (mg) | 200.0 | 0.0 | 0.0 | 0.0 | 0.0 |
| Niacin (mg) | 10.0 | 13.9 | 25.8 | 11.9 | 35.6 |
| Folate (µg) | 300.0 | 3.6 | 0.0 | 6.0 | 10.8 |

**Abbreviations:** UL, upper limit defined according to European Food Safety Authority values; YCF, young child formula.
